# 'BeSAFE', effect-evaluation of internet-based, tailored safety information combined with personal counselling on parents' child safety behaviours: study design of a randomized controlled trial

**DOI:** 10.1186/1471-2458-10-466

**Published:** 2010-08-09

**Authors:** Mirjam EJ van Beelen, Tinneke MJ Beirens, Mirjam K Struijk, Paul den Hertog, Anke Oenema, Eduard F van Beeck, Hein Raat

**Affiliations:** 1 Department of Public Health, Erasmus MC - University Medical Centre Rotterdam, PO BOX 2040, 3000 CA Rotterdam, the Netherlands; 2 Consumer Safety Institute, PO BOX 75169, 1070 AD Amsterdam, the Netherlands

## Abstract

**Background:**

Injuries in or around the home are the most important cause of death among children aged 0-4 years old. It is also a major source of morbidity and loss of quality of life. In order to reduce the number of injuries, the Consumer Safety Institute introduced the use of Safety Information Leaflets in the Netherlands to provide safety education to parents of children aged 0-4 years. Despite current safety education, necessary safety behaviours are still not taken by a large number of parents, causing unnecessary risk of injury among young children. In an earlier study an E-health module with internet-based, tailored safety information was developed and applied. It concerns an advice for parents on safety behaviours in their homes regarding their child. The aim of this study is to evaluate the effect of this safety information combined with personal counselling on parents' child safety behaviours.

**Methods/Design:**

Parents who are eligible for the regular well-child visit with their child at child age 5-8 months are invited to participate in this study. Participating parents are randomized into one of two groups: 1) internet-based, tailored safety information combined with personal counselling (intervention group), or 2) personal counselling using the Safety Information Leaflets of the Consumer Safety Institute in the Netherlands for children aged 12 to 24 months (control group). All parents receive safety information on safety topics regarding the prevention of falling, poisoning, drowning and burning. Parents of the intervention group will access the internet-based, tailored safety information module when their child is approximately 10 months old. After completion of the assessment questions, the program compiles a tailored safety advice. The parents are asked to devise and inscribe a personal implementation intention. During the next well-child visit, the Child Health Clinic professional will discuss this tailored safety information and the implementation intention with the parents. The control group will receive usual care, i.e. the provision of Safety Information Leaflets during their well-child visit at the child's age of 11 months.

**Discussion:**

It is hypothesized that the intervention, internet-based, tailored safety information combined with personal counselling results in more parents' child safety behaviours.

**Trial registration:**

Current Controlled Trials NTR1836

## Background

Injuries in or around the home are the most important cause of death among children aged 0-4 years old. It is also a major source of morbidity and loss of quality of life [1-3]. In the Netherlands each year 30 children aged 0-4 years die caused by injuries in or around the home. Additionally 57.000 children aged 0-4 years are medically treated, of which 46.000 children at the emergency room of a hospital because of home injuries [4]. In order to reduce the number of injuries, the Consumer Safety Institute introduced the use of Safety Information Leaflets in the Netherlands to provide safety education to parents of children aged 0-4 years. These leaflets are well used in Child Health Clinics (CHC) and indications for a small effect on parental behaviours were gained through observational research [5,6]. However, despite current safety education, necessary safety behaviours are still not taken by a large number of parents, causing unnecessary risk of injury of young children. Improving the effectiveness of safety education to parents at CHC is therefore desirable. In an earlier study an E-health module with internet-based, tailored safety information was developed and applied. It concerns an internet-based, tailored information in combination with personal counselling for parents of infants on safety behaviours to be taken to the homes for their child [7-9]. In a process-evaluation it was found that majority of parents experience the new internet-based, tailored safety information as useful and applicable and that the CHC professionals are enthusiastic about the E-health module [8]. However there are no insights in the effects of the new internet-based, tailored safety information on parents' child safety behaviours compared to the current way of safety education.

### Objectives

The objective of this study is to evaluate the effect of online, internet-based, tailored safety information combined with personal counselling on parents' child safety behaviours. Additionally a process evaluation will be conducted to provide insight in the feasibility of the intervention. In this article the design of the study is described.

### Study hypothesis

The hypothesis of the study is that, after follow-up, parents of the intervention group show more safety behaviours regarding the prevention of falling, poisoning, drowning and burning compared to the control group. Furthermore we hypothesize that, determinants of safe behaviour, i.e. severity and self efficacy positively improve in the intervention group [10-14].

## Methods/design

### Study design

The study design is a randomized controlled trial (RCT), with a baseline measure point prior to the intervention and a follow-up measure point six months after the intervention. The course of the study with the specific items at each time point is described in table 1. Parents are individually randomized in an intervention group or a control group, according to a computerized random allocation generator. Parents had an equal probability of assignment to the groups. Parents of the intervention group receive internet-based, tailored safety information concerning the prevention of falling, poisoning, drowning and burning, combined with personal counselling at the CHC. Parents of the control group receive 'care as usual', personal counselling at the CHC using the Safety Information Leaflets (children 12-24 months old) of the Consumer Safety Institute in the Netherlands, concerning the same four safety topics.

**Table 1:** Course BeSAFE study

Age of the child	Intervention-group	Control-group
**5-8 months**	▪ Informing parents about the study ▪ Request for participation (max. 2 reminders) ▪ Information letter ▪ Information folder ▪ Questionnaire	▪ Informing parents about the study ▪ Request for participation (max. 2 reminders) ▪ Information letter ▪ Information folder ▪ Questionnaire

**5-8 months**	▪ Parents log in on the website ▪ Parents provide informed consent ▪ Parents complete the **Baseline Questionnaire**	▪ Parents log in on the website ▪ Parents provide informed consent ▪ Parents complete the **Baseline Questionnaire**
		
	Randomization in intervention or control group	
		
**10 months**	Parents are invited through e-mail to complete the **online, internet-based, tailored safety information questionnaire **(max. 2 reminders)	

**11 months**	**Well-child visit** ▪ Personal counselling to discuss the **internet-based, tailored safety information and implementation-intention plan** ▪ Complete process-evaluation form by parents ▪ Complete process-evaluation form by Child Health Care professional	**Well-child visit** ▪ Usual Care: Safety Information Leaflets

**12 months**	Repeating the **internet-based, tailored safety information and implementation intention**, send to the parent by e-mail	

**17 months**	**Follow-up questionnaire** Follow up questionnaire send to the parent (max. 2 reminders)	**Follow-up questionnaire** Follow up questionnaire send to the parent (max. 2 reminders)

Data collection started in 2009 and will continue until 2011. This study is approved by the Medical Ethics Committee of Erasmus MC (MEC-2008-370).

### Study procedure and participants

Parents who are eligible for the regular well-child visit with their child at child age 7.5 months receive written information about the study and are invited to provide informed consent to participate in the study. All parents receive a singular, personal code to log in at the website of the study (www.besafe-onderzoek.nl). In 2009, 90% of all households in the Netherlands had access to the internet [15]. At the study website parents can find more information about the study and they can complete the questionnaires.

#### Youth Health Care organisations and Child Health Clinic teams

Managers of an opportunity sample of 26 Youth Health Care (YHC) organisations in the Netherlands were informed about the study and were contacted by the researchers to provide further information. Five YHC organisations in the provinces of Zuid-Holland, Noord-Brabant and Zeeland volunteered to participate in the study, with a total of 30 CHC teams. These teams cover both urban and rural regions in the Netherlands. Prior to the start of the study, the researchers arranged meetings to explain the procedure of the study and to instruct the participating CHC professionals.

#### Children and their parents or caregivers

The study population consists of parents or caregivers of toddlers (one per family). They are included in the study when their child is 5-8 months old and measurements continue until the child is circa 17 months old. Parents of children in the age range of this study have a high attendance percentage (90%) at Child Health Clinics [15].

**Figure 1:**
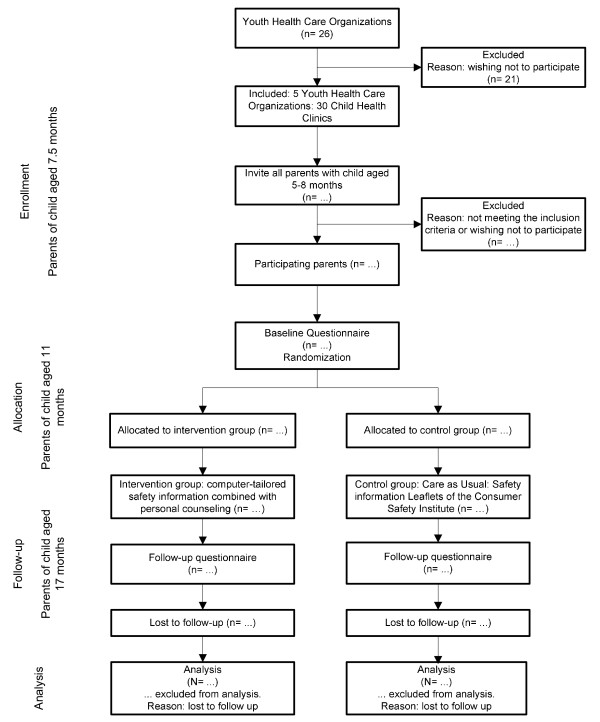
**Flow chart of the participants and allocation through the trial**.

Only parents who understand the Dutch language and have access to the internet are eligible to be included in the study. The study design and participant flow are shown in Figure 1.

### Intervention

The BeSAFE intervention aims at 4 major topics on safety in or around the home of children aged 12 to 24 months; prevention of falling, poisoning, drowning and burning [5,8,16-22]. The different components of the safety topics of the intervention can be found in table 2. The intervention is based on the social ecological model, where safety and health are influenced by a combination of environment (in and around the home) and personal factors (the parents) and the interaction between them [23]. Parents are the most important mediators of the environment of these young and vulnerable children. Parents influence both the physical environment as the social environment of the child, mediated by the parenting style and specific parenting practices [24-27]. Therefore interventions should be aimed at the parents to guarantee the safety and health of the young child. Determinants of parents' behaviours can be found in the Protection Motivation Theory (PMT) and the theory of planned behaviour and includes severity, vulnerability, response efficacy, self efficacy and intentions [10,12-14].

**Table 2:** Safety advices of the intervention based on safety behaviour in and around the home

Safety behaviour concerning the prevention of	Applicable if:	Reinforcement or NO information when:
**Falling**		
- Stair gate	- The house has a staircase which the child can reach	- A stair gate is present en is being used at all times
- Balcony	- The house has a balcony	- The child is never left alone on the balcony
		
**Poisoning**		
- Cleaning products	- Always	- Stored in a closet with a lock or higher than 1.50 meters
- Medicines	- Always	- Stored in a closet with a lock or higher than 1.50 meters
		
**Drowning**		
- Bath tub	- The child takes a bath	- Never left alone in the bath tub
- Swimming pool	- The child swims in the swimming pool	- Never left alone in the swimming pool
- Pond	- There is a pond in the garden	- Always the information to fill up the pond
		
**Burns**		
- Thermostat-controlled tap	- Always	- Thermostat-controlled tap present
- Hot drinks	- Always	- Child never on parents womb when drinking hot drinks
- Kitchen	- Always	- Child never in the kitchen when cooking

The BeSAFE intervention consists of internet-based, tailored safety information combined with personal counselling at the CHC. The BeSAFE intervention module consists of a questionnaire assessing ten specific parents' child safety behaviours in the prevention of falling, poisoning, drowning and burning and determinants of safety behaviour (intentions, possible barriers and self efficacy); a library of safety messages; and algorithm to compose tailored health information for the parent [28]. Parents who give informed consent and fill in the baseline questionnaire receive an e-mail, when their child is about 10 months old, inviting them to participate in the BeSAFE intervention. As a first step parents complete a questionnaire, which is used to generate an online tailored safety advice. When parents have read their personal advice, they are invited to devise and inscribe an implementation intention. In this implementation intention parents plan specific actions for improving their safety behaviour and implementing them in their home situation at a specified time [29,30]. It consists of three parts, i.e. what, when and where. The safety information and implementation intention of each parent will be sent by e-mail to both the parent and the CHC professional, in order to prepare them for the well-child visit at 11 months. At the well-child visit the CHC professional will discuss the safety information and the implementation intention with the parent, using the techniques of motivational interviewing (MI). Motivational interviewing is believed to represent a brief and effective method for addressing behaviour change. Positive attitudes and experienced barriers of the parents are discussed in order to improve parents' child safety behaviours. One aspect of this motivational interviewing is giving attention to transforming positive intention into real behaviour [29-31]. CHC professionals were trained to apply motivational interviewing. Approximately four weeks after the well-child visit parents receive a reminder of their safety information and the implementation intention in order to strengthen the message.

### Control

The control group will receive care us usual; i.e. parents receive a Safety Information Leaflet (for children aged 12-24 months) of the Consumer Safety Institute during their CHC visit at the child age of approximately 11 months [6,21,22,32]. The Safety Information Leaflets contain relevant information about the prevention of injuries of toddlers in or around the home, divided in general information and safety advices about the prevention of falling (i.e. window protection, stair gates, practice walking down the stairs), poisoning (i.e. safe storage of cleaning products and medicines), drowning (i.e. ponds) and burning (i.e. hot fluids, hot pans) [22].

## Measurements

### Primary outcome measures

The primary outcomes of the study are parents' child safety behaviours measured at the child's age of 17 months, regarding the prevention of falling, poisoning, drowning and burning, i.e. presence and use of stair gates, never leaving the child alone on the balcony, safe storage of cleaning products and medicines, never leaving the child alone in the bath tub, safety of a swimming pool or a pond in the garden, thermostat controlled taps, drinking hot fluids while the child is on the parent's lap and keeping the child out of the kitchen while the parents is cooking. In the questionnaires parents are asked which safety behaviours they take in their homes. Some behaviours are only assessed when they are applicable to the situation of the parent. For example, when there are no stairs in the homes, no questions about installing stair gates will be asked.

Presence of safety measures, i.e. stair gate or thermostatic controlled taps is defined as present/not present. Safety behaviour, i.e. closing the stair gate, storing cleaning products after use and drinking hot fluids with a child on parent's lap is scored on a five-point scale from 'never' to 'always'.

### Secondary outcome measures

The secondary outcomes are the determinants of the above mentioned parents' child safety behaviours, i.e. severity, vulnerability, response efficacy, self efficacy and intentions. Secondary outcomes, except intentions, are measured on five-point Likert scales.

Severity is measured with one item per safety measure, asking how seriously they perceived the consequences of this event (from not serious at all to very serious). Vulnerability is measured by asking respondents their perception of their child's risk of an unintentional injury on each specific subject (from low risk to high risk).

Response efficacy is assessed by asking how helpful parents perceived the specific behaviour to be for preventing an injury (from very helpful to not very helpful).

Self efficacy is measured by asking parents how difficult or easy they perceive taking the safety measures to be (from very easy to very difficult).

Intentions are assessed by asking whether the parent intends to take the specific safety measure. Answers to be given are yes, within one month; yes, within one to six months, yes, but not within six months; or no intention.

### Baseline questionnaire

The baseline questionnaire, completed at child age of circa 7, 5 months, consists of questions on pregnancy, birth, gender, ethnicity of the child and the parents, educational level of the parents, household and family composition, the ten specific parents' child safety behaviours (presence and use of stair gates, never leaving the child alone on the balcony, safe storage of cleaning products and medicines, never leaving the child alone in the bath tub, safety of a swimming pool or a pond in the garden, thermostat controlled taps, drinking hot fluids while the child is on the parent's lap and keeping the child out of the kitchen while the parents is cooking) and the determinants of these safety behaviours (severity, vulnerability, response efficacy, self efficacy and intentions).

### Follow-up questionnaire

When the child is approximately 17 months old, 6 months after the intervention, all participating parents will receive a follow-up questionnaire. This questionnaire contains the same items on safety behaviours and the determinants of these safety behaviours.

### Process-evaluation

In addition to the effect-evaluation a process-evaluation will be carried out. All parents who use the BeSAFE intervention module are asked to answer a few evaluating questions about the programme, i.e. which part of the advice parents have read, what there opinion is about the advice, do parents intend to change anything in there behaviour after reading the advice and what they think about the time they needed to complete the module. All parents in the intervention group and CHC professionals who provide the intervention will be asked to complete a process-evaluation form after the well-child visit at 11 months where the tailored safety information is discussed. It consists of questions regarding the feasibility of the intervention within the well-child visit, the perceived usefulness of the intervention and the discussed items during the well-child visit.

### Power of the study

We will calculate sum scores of parents' child safety behaviours (0-10 points) of all participating parents, at follow-up as well as at baseline. Power calculations showed that a total number of 1200 parents are needed to detect a difference of 0.34 points between intervention and control group, assuming a mean score of 3.5 points and a standard deviation of 1.7 points, with a power of 0.80 and alpha 0.05. Assuming a participation of 50% and a loss-to-follow-up of 30%, we will have complete data at follow-up form 840 parents (420 in both the intervention and control group).

Considering the dichotomous outcome measures of 'stair gate present' we assume an unsafe situation in 30% of families in the control group [8]. A difference of 9% between the percentages unsafe families of the intervention group and the control group can be shown (21% in the intervention group, 30% in the control group).

Considering the dichotomous outcome measures of 'safe storage of cleaning products' we assume an unsafe situation in 20% of families in the control group [33]. A difference of 8% between the percentages unsafe families of the intervention group and the control group can be shown (12% in the intervention group, 20% in the control group).

### Statistical analyses

Statistical analyses are performed using SPSS 16.0 (SPSS Inc., Chigaco, IL.)

Descriptive statistics are used to describe parents and child characteristics and variable scores (behaviours) for the intervention and control group at baseline and follow-up.

#### Effect-evaluation

The aim of the study is to assess the effect of internet-based, tailored safety information combined with personal counselling on parents' child safety behaviours. An intention-to-treat analysis will be applied [34]. Regression analysis will be used to evaluate continuous outcome (sum scores) variables, with group (intervention or control group) as independent variable and the baseline values as covariates. Logistic regression analysis will be performed for the evaluation of dichotomous outcomes. Additionally effect modification by composition of the family (one versus two children), educational level and ethnicity of the parents will be explored.

#### Process-evaluation

In addition to the effect-evaluation a process-evaluation will be carried out. Adherence of both the CHC professionals and parents to the different elements of the BeSAFE intervention will be evaluated [35].

## Discussion

This article describes the design of a randomised controlled trial regarding the BeSAFE intervention intended to promote parents' child safety behaviours. The study evaluates the effect of internet-based, tailored safety information combined with personal counselling on parents' child safety behaviours. We want to look at parents' child safety behaviours and want to compare these behaviours between the intervention and the control group. The new elements which are applied in the intervention group include a tailored safety advice for the parent, an implementation intention filled in by the parent and the discussion of this advice and implementation intention by the CHC professional with the parents using the techniques of motivational interviewing.

It is hypothesized that after 6 months of follow-up, parents in the intervention group show more child safety behaviour regarding the prevention of falling, poisoning, drowning and burning. Differences between subgroups (ethnicity and socio-economic status) regarding the effects of the intervention will be explored.

Strengths of the study are the power of the study, the randomized controlled design, and providing the intervention in daily practice of the CHC, which have a high attendance. The follow-up at 6 months allows investigating the effect of the intervention within an appropriate time schedule in the development of the child. Regarding the generalisability of the study results there can be noticed that it is a randomized controlled study conducted in the practice setting. The intervention is applicable in daily practice of the CHC professional, which will facilitate the implementation of the internet-based, tailored safety information if it is found effective. The data will be collected in both rural and urban areas of the Netherlands, resulting in higher generalisability.

Because the study relies on self-report by parents, misclassification might occur. Parents might give socially desirable answers by overstating their safety behaviours. A limitation of the study to be addressed includes the questionnaire and intervention being available in Dutch only. For this reason it is likely that only parents who master the Dutch language will participate in the study.

In conclusion, this study evaluates the effect of internet-based, tailored safety for parents of young children, combined with personal counselling at the Child Health Clinic.

## Competing interests

The authors declare that they have no competing interests.

## Authors' contributions

HR had the original idea for the study and its design, and was responsible for acquiring the study grant. MB is responsible for the data collection, data analysis and reporting of the study results. MS helps coordinate the study and participates in data collection.

EB, PH, MS, TB and AO provide expert input during the study. HR and TB supervise the study. All authors regularly participated in discussing the design and protocols used in the study. All authors read and approved the final manuscript.

## Pre-publication history

The pre-publication history for this paper can be accessed here:

http://www.biomedcentral.com/1471-2458/10/466/prepub
